# Impact of the ´Alforja Educativa’ on Ecuadorian schoolchildren’s knowledge of bacteria, antibiotics, and antibiotic resistance, a pretest-posttest study

**DOI:** 10.1186/s12889-024-18340-0

**Published:** 2024-03-18

**Authors:** Ambar Célleri-Gomezcoello, Caroline Avila, Silvina Alessio, Celina M. Hanson, Andrea Ochoa, Miriann Mora, Liliana Arciniegas, Bronwen Holloway, Maria Pränting, Daniela Encalada

**Affiliations:** 1 Universidad del Azuay, Av. 24 de Mayo 7-77, Cuenca, 010107 Ecuador; 2 ReAct Latin America, Fundación Niño a Niño, Tomás Ordóñez 9-18 y Simón Bolívar, Cuenca, 010101 Ecuador; 3 ReAct Europe, Department of Medical Sciences, Uppsala University, Box 256, Uppsala, 751 05 Sweden; 4 Facultad de Medicina, Universidad Católica de Cuenca, Av. de las Américas y Humboldt, Cuenca, 010105 Ecuador

**Keywords:** Antibiotic resistance, Antimicrobial resistance, Education, One health, Bacteria, Antibiotics, Microbiome, Microbial world

## Abstract

**Background:**

Widespread use of antibiotics disrupts the balance in the microbial world and promotes development and spread of antibiotic resistant bacteria. Educational initiatives are important as part of strategies to mitigate antibiotic resistance. The Alforja Educativa is an innovative educational program developed in Ecuador with the aim to teach schoolchildren about antibiotic use and antibiotic resistance. The program places antibiotic resistance within a broader frame of health, well-being, and ecological awareness, highlighting the importance to maintain balance in the microbial world. The objective of this study was to evaluate the effect of the Alforja Educativa on knowledge about bacteria, antibiotics and antibiotic resistance amongst fifth and sixth grade Ecuadorian schoolchildren.

**Methods:**

This pretest-posttest intervention study was conducted between April and June 2017 and comprised fifth and sixth grade schoolchildren from 20 schools in Cuenca, Ecuador, recruited by purposeful sampling. The Alforja Educativa was implemented over twelve 80-minute sessions by trained university students. Schoolchildren’s knowledge was assessed before and after participation in the educational program using a structured questionnaire. A mean total score, the proportion of correct responses for each individual knowledge-based question, as well as correct responses for each of the multiple-choice options of the knowledge-based questions were calculated for the pretest and posttest.

**Results:**

A total of 1,257 schoolchildren participated in the Alforja Educativa program, of which 980 (78%) completed both the pretest and posttest. Overall, the mean total knowledge score increased from pretest to posttest (2.58/7.00 vs. 3.85/7.00; CI = 0.5, *p* < 0.001). After participation in the program, the proportion of schoolchildren that correctly identified that bacteria can be both good and bad increased from 35.0 to 84.3%. In addition, scores increased for correctly identifying the meaning of antibiotic resistance (37.4–72.0%); how to prevent antibiotic resistance (63.2–74.6%); and for identifying the meaning of self-medication (46.3–54.3%).

**Conclusion:**

The Alforja Educativa was effective in improving the knowledge of participating schoolchildren about concepts related to bacteria, antibiotics and antibiotic resistance. The holistic perspective taken to explain the complex relationship between humans and bacteria, as well as the effect of antibiotics on the microbial world, may help provide a foundation for more sustainable antibiotic use.

**Supplementary Information:**

The online version contains supplementary material available at 10.1186/s12889-024-18340-0.

## Background

Antibiotics are medicines used to kill or stop the growth of bacteria in order to prevent and treat infections in humans and animals. However, only a few select bacteria are responsible for causing disease, with the majority of bacteria playing a beneficial role, performing vital functions for the host organisms where they co-exist, as well as in the environment [1]. The use of antibiotics affects not only disease-causing bacteria, but also other harmless and important bacteria. While antibiotics are important, life-saving medicines, all use of antibiotics disrupts the balance in the microbial world and promotes the development and spread of antibiotic resistant bacteria [2]. Already, an estimated 1.27 million people globally die each year from antibiotic resistance, with the highest burden falling on low- and middle-income countries [3]. Widespread use of antibiotics when not indicated unnecessarily fuels this process.

Context-appropriate interventions to improve the use of antibiotics are needed to maintain the health of the microbial world and slow the development of antibiotic resistance [4]. The Global Action Plan on antimicrobial resistance stresses the importance of education about antibiotic use and resistance, and advocates for the inclusion of these topics in school curricula to promote better understanding and awareness from an early age [5]. Educating schoolchildren on the importance of bacteria for the health of humans, animals and the environment, as well as antibiotics and their effect on the microbial world, can provide a foundation for more sustainable antibiotic use [6, 7].

The Alforja Educativa: Student Health and the Microbial World, is an innovative educational program to promote health and ecological awareness, with a specific focus on bacteria, antibiotics and antibiotic resistance, targeted at audiences in Latin America [8, 9]. The educational program is grounded in the indigenous concept of Sumak Kawsay, a philosophy of life that recognizes the interdependence and interrelation of all beings, and promotes living in harmony within communities, with oneself, and with nature [10]. The Alforja Educativa builds on these native ancestral principles to promote a paradigm shift in youth health education that takes a more holistic perspective to explain the complex relationship between humans and bacteria. Thus, highlighting the importance to use antibiotics with care to maintain the balance in the microbial world and limit the development of antibiotic resistance.

Conceptualization of the Alforja Educativa began in 2012 in Cuenca, Ecuador in a joint effort between ReAct– Action on antibiotic resistance and the Child to Child Center. First a review of the local school curriculum and study materials was carried out, including interviews and conversations with teachers. During this process, it was found that bacteria were rarely mentioned and always in relation to their role in disease. Antibiotic resistance was not a topic within the school curriculum. The Alforja Educativa was then developed by an interdisciplinary and inter-institutional team composed of teachers, health and education professionals, artists, writers and communicators, and was co-developed with schoolchildren through an iterative educational training and review process [8, 9]. The Alforja Educativa promotes experiential learning of science using the child-to-child methodology, an educational approach that links schoolchildren’s learning with taking action to promote the health, well-being and development of not only themselves, but also their families and the communities within which they live [11, 12]. The Alforja Educativa consists of illustrated activity guides, supported by graphic, digital and audio-visual teaching materials such as songs, videos, stories, and comics [8, 9, 13]. It has been developed towards the fourth to sixth grade education level, based on the schoolchildren’s cognitive level, literacy skills, and the child-to-child methodology, however the content is flexible and can be adapted to different grades. The objective of this study was to evaluate the effect of the Alforja Educativa on knowledge of fifth and sixth grade Ecuadorian schoolchildren about bacteria, antibiotics, and antibiotic resistance.

## Methods

### Study design, setting and participants

A pretest-posttest intervention study was conducted to measure the knowledge about bacteria, antibiotics and antibiotic resistance among schoolchildren that participated in the Alforja Educativa. Eighty university students studying medicine or education in their second year of pre-professional education at Universidad del Azuay and Universidad Católica de Cuenca were recruited to implement the Alforja Educativa and perform the data collection.

The study took place at 20 elementary schools in the surrounding area of Cuenca, Ecuador. Schools were purposively selected that were pre-professional practice centers for the two universities, and based on their proximity to Cuenca. Of the 20 participating schools, 17 were public and three were private. Ten were located in urban areas, and ten in rural areas. Fifth and sixth grade schoolchildren participated in the educational program. In Ecuador this is predominately children of ages 9–10 years, however students may also be a few years younger or older depending on the setting.

### Intervention

The project team developed a guide for university students to follow when implementing the Alforja Educativa. The university students were trained on the guide through three sessions of four hours each in March 2017. The training included concepts of the microbial world, use of antibiotics, antibiotic resistance, Sumak Kawsay, the child-to-child methodology, and the application of the modules to schoolchildren, as well as information about the pretest-posttest study design. In addition, the medical students were trained on how to develop classes and learning cycles from didactics, and education students were trained on medical content and how to teach in a workshop mode. The trained university students were divided into groups of two to four and paired with schools based on their previous experience with the communities in which the schools were based. The teams consisted of either education or medical students, and the students did not change schools after pairing.

The participating schoolchildren were briefed on the Alforja Educativa, the implementation process and study by their regular teachers and the university students before the program began. The Alforja Educativa was then implemented in the normal class schedule where the regular teachers left the classrooms, and the university students came into the classrooms for 80-minute sessions that occurred once a week over a period of 12 weeks between April and June 2017. The sessions covered the themes of the microbial world, Sumak Kawsay, appropriate use of antibiotics and antibiotic resistance. The Alforja Educativa activity guides and other supportive materials [8, 9, 13] were used by the university students to help plan lectures, classroom activities, homework assignments and reflective exercises together with the schoolchildren. Key learning objectives included (i) to understand Sumak Kawsay as harmony between all beings and mother nature; (ii) to change the negative and fearful perception we have of bacteria to instead perceive them as beings that play a beneficial role for us and the ecosystem; (iii) to understand how we can live in harmony with microbes and what is important to maintain the health of the ecosystem, children and the community; (iv) to describe the relationship between the use of antibiotics and antibiotic resistance; (v) to understand the importance of appropriate use of antibiotics and; (vi) to be able to communicate about bacteria, antibiotics and antibiotic resistance at home, school, and in the community.

### Data collection

The pretest was administered by the university students before they began the Alforja Educativa program. After conducting the 12 sessions, the schoolchildren were given the posttest (after approximately 3 months). The children answered on a voluntary basis with no controlling if the children completed the questionnaires. The pretest and posttest were in written form and contained the same set of eight multiple-choice questions in Spanish, Q1-Q8. One question (Q4), asked about the schoolchildren’s experiences. Seven questions were used to measure the schoolchildren’s knowledge on the topics of bacteria (Q2, Q3), antibiotics and the appropriate use of medicines (Q6, Q7), antibiotic resistance (Q5, Q8), and Sumak Kawsay (Q1). For two questions (Q3, Q6), multiple answers were correct, while the remaining questions had only one correct answer (Q1, Q2, Q5 and Q7). For the purpose of the publication all questions were translated into English (see Additional file 1 for translated questionnaire).

### Data analysis

Data from schoolchildren that completed both a pretest and a posttest were considered eligible for analysis in this study. Demographic measures included age, gender (boys or girls), school type (public or private), and geographical area of school (urban or rural). Total pretest and total posttest scores were calculated for each schoolchild based on the number of correct answers to knowledge-based questions (range = 0 to 7, where 7 was the highest score). Schoolchildren’s responses were considered correct if they had chosen the correct multiple-choice option(s) and no other options. A mean total score for the entire population was then calculated for the pretest and posttest. In addition, the proportion of correct responses was calculated for each individual knowledge-based question, as well as for each of the multiple-choice options of the knowledge-based questions. The change in total score from pretest to posttest was also calculated for each schoolchild, and coded to represent if there was a decrease, increase or no-change, in order to allow analysis of association with demographic variables.

Descriptive statistics were used for demographic data, Q4 (about the schoolchildren’s experiences), the individual knowledge-based questions, as well as the multiple-choice options of the knowledge-based questions. To evaluate changes from pretest to posttest, paired ’t’ test was used to compare the difference in mean total score, and McNemar’s test was used to compare the proportion of correct responses to individual knowledge questions, as well as their respective multiple-choice options. Chi-square test was applied to examine the association of change in total score from pretest to posttest with the demographic variables. A *p*-value of < 0.05 was considered significant in this study. Data analyses were conducted using IBM^®^ SPSS^Ⓡ^ Statistics 28 and STATA (version 17; Stata Corp., College Station, TX, USA).

## Results

A total of 1,257 schoolchildren took the pretest and 1,201 the posttest. However, the total number of schoolchildren that were eligible for data analysis, having completed both the pre- and posttest, was 980 (78%). The number of eligible schoolchildren per school ranged from 11 to 154 with an average of 49. Baseline characteristics of schoolchildren is provided in Table 1. The median age of schoolchildren was ten years. In private schools ages ranged from 9 to 11, while in public schools the range was 7–13. Overall, 55.8% were girls and 44.2% were boys. The majority of children, 78.4% (*n* = 768), were from public schools, and the remaining 21.6% (*n* = 212) were from private schools. In the rural area, all 480 schoolchildren attended public schools. Of the 500 schoolchildren from the urban area, 57.6% (*n* = 288) attended public schools and 42.4% (*n* = 212) private schools. There was no significant difference in the proportion of girls versus boys at private schools (59.7% [172/288] vs. 54.7% [116/212], *p* = 0.263).

**Table 1:** Baseline characteristics of participating schoolchildren (*N* = 980)

Characteristic	*n*	(%)
Age (years)		
7	1	(0.1)
8	4	(0.4)
9	389	(39.7)
10	468	(47.8)
11	106	(10.8)
12	9	(0.9)
13	3	(0.3)
Gender		
Girls	547	(55.8)
Boys	433	(44.2)
School Type		
Public	768	(78.4)
Private	212	(21.6)
Geographic Setting		
Urban	500	(51.0)
Rural	480	(49.0)

Q4 asked schoolchildren where they remembered to have seen or heard something about bacteria. Of them, 58.8% (*n* = 576) responded in school, 48.8% (*n* = 478) at home, 47.4% (*n* = 465) from TV, 28.1% (*n* = 275) from radio, and 12.9% (*n* = 126) responded at supermarkets. Of all the schoolchildren, 4.9% (*n* = 48) marked that they had not previously heard of bacteria anywhere.

The mean total score increased significantly from pretest to posttest (2.58/7.00 [36.9%] vs. 3.85/7.00 [55.0%] CI = 0.5, *p* < 0.001). A significant increase was observed in the proportion of schoolchildren who correctly answered each individual knowledge-based question except for Q6 (Table 2). The greatest increase was for the identification of what bacteria are like (good and bad, Q2), which increased from 35.0 to 84.3% pretest to posttest. Following this, scores increased from pretest to posttest for correctly identifying: the meaning of antibiotic resistance (37.4–72.0%, Q5); the meaning of “Sumak Kawsay” (62.9–79.2%, Q1); how to prevent antibiotic resistance (63.2–74.6%, Q8); the meaning of self-medication (46.3–54.3%, Q7); and that bacteria serve to produce vitamins and are important to life (1.9–8.4%, Q3).

Across the seven individual knowledge-based questions there were a total of 32 multiple-choice options, excluding “other” which almost no schoolchildren chose. The proportion of schoolchildren that correctly answered the individual multiple-choice options at pretest and posttest (that is, that correctly picked or did *not* pick a specific option), as well as the changes from pretest to posttest are described below and shown in Table 2. For 23 of the options there was a significant positive change, while three had a significant negative change in relation to the learning objectives of the Alforja Educativa program. Note that even though schoolchildren may have picked the correct option, they may have incorrectly chosen other options within the same question, thus not scoring correctly on the overall question. Before beginning the program, 71.8% of the schoolchildren picked the correct answer for the meaning of Sumak Kawsay (Q1), this increased to 82.7% at posttest. For Q2, schoolchildren that answered that bacteria can be both good and bad, and likewise, that did *not* answer that bacteria are only bad, increased after participation in the Alforja program (37.0-85.7% and 42.2–96.0%, respectively). Similarly, in Q3, more schoolchildren understood that bacteria do not only serve to make us sick (22.4–89.8%), and that bacteria are important to life (13.9–69.6%). For Q6 there were mixed results. More schoolchildren correctly answered that antibiotics should not be used to treat a cold nor stopped as soon as the person feels better (71.8–87.3% and 72.8–93.1%, respectively). However, in the same question, less schoolchildren correctly identified that antibiotics cure diseases caused by bacteria (58.5–31.5%). For Q7, more schoolchildren understood that self-medication is when we take medicine without going to the doctor (48.9–57.8%), and it is not when the doctor prescribes us remedies (68.2–73.5%). For Q5, the proportion of schoolchildren who correctly identified the meaning of antibiotic resistance increased from 41.6 to 74.5%. Finally, for Q8, the majority of schoolchildren already answered correctly at pretest how to prevent antibiotic resistance, still modest positive improvements were seen for several of the multiple-choice options.

**Table 2:** Proportion of correct responses to questions, multiple-choice options, and change from pretest to posttest (*N* = 980)

	Pretest		Posttest		Change		
	*n*	(%)	*n*	(%)	*n*	(%)	*p*-value
**Q1. What does “Sumak Kawsay” or “Good Living” mean? Mark with an X what you think is correct**:							
To have everything you need for life.	786	(80.2)	868	(88.6)	82	(8.4)	< 0.001
To live in harmony between human beings and nature.*	704	(71.8)	810	(82.7)	106	(10.8)	< 0.001
To have a lot of money.	952	(97.1)	957	(97.7)	5	(0.5)	0.4838
I don’t know.	854	(87.1)	912	(93.1)	58	(5.9)	< 0.001
**Schoolchildren chose the correct option and no other option(s)**	**616**	**(62.9)**	**776**	**(79.2)**	**160**	**(16.3)**	**< 0.001**
**Q2. Mark with an X the drawing that represents what bacteria are like**:							
Good	902	(92.0)	854	(87.1)	-48	(-4.9)	< 0.001
Good and Bad*	363	(37.0)	840	(85.7)	477	(48.7)	< 0.001
Bad	414	(42.2)	941	(96.0)	527	(53.8)	< 0.001
**Schoolchildren chose the correct option and no other option(s)**	**343**	**(35.0)**	**826**	**(84.3)**	**483**	**(49.3)**	**< 0.001**
**Q3. Mark with an X the correct answers**:							
Bacteria produce vitamins.*	62	(6.3)	227	(23.2)	165	(16.8)	< 0.001
Bacteria are important for life.*	136	(13.9)	682	(69.6)	546	(55.7)	< 0.001
Bacteria only serve to make us sick.	220	(22.4)	880	(89.8)	660	(67.3)	< 0.001
I don’t know.	890	(90.8)	899	(91.7)	9	(0.9)	0.4517
**Schoolchildren chose the correct options and no other option(s)**	**19**	**(1.9)**	**82**	**(8.4)**	**63**	**(6.4)**	**< 0.001**
**Q5. What do you think antibiotic resistance means? Mark with an X the correct answer**:							
That bacteria are resistant to alcohol.	829	(84.6)	893	(91.1)	64	(6.5)	< 0.001
That bacteria are resistant to soaps.	798	(81.4)	906	(92.4)	108	(11.0)	< 0.001
That bacteria are resistant to antibiotics.*	408	(41.6)	730	(74.5)	322	(32.9)	< 0.001
I don’t know.	724	(73.9)	880	(89.8)	156	(15.9)	< 0.001
**Schoolchildren chose the correct option and no other option(s)**	**367**	**(37.4)**	**706**	**(72.0)**	**339**	**(34.6)**	**< 0.001**
**Q6. Mark with an X what you consider correct about the use of antibiotics. It is possible to indicate more than one option**:							
Antibiotics are bought without a prescription.	881	(89.9)	895	(91.3)	14	(1.4)	0.2714
Antibiotics should always be prescribed by the doctor.*	617	(63.0)	671	(68.5)	54	(5.5)	0.0034
Antibiotics are remedies that help you grow.	863	(88.1)	902	(92.0)	39	(4.0)	0.0024
Antibiotics are remedies to cure illnesses caused by bacteria.*	573	(58.5)	309	(31.5)	-264	(-26.9)	< 0.001
You can take antibiotics to treat a cold.	704	(71.8)	856	(87.3)	152	(15.5)	< 0.001
Antibiotics should be taken as prescribed by the doctor.*	491	(50.1)	447	(45.6)	-44	(-4.5)	0.0218
Antibiotics should be stopped as soon as the person feels better.	713	(72.8)	912	(93.1)	199	(20.3)	< 0.001
I don’t know.	836	(85.3)	889	(90.7)	53	(5.4)	< 0.001
**Schoolchildren chose the correct options and no other option(s)**	**115**	**(11.7)**	**122**	**(12.4)**	**7**	**(0.7)**	**0.6197**
**Q7. What does self-medication mean to you? Mark with an X the correct answer**:							
It is when we drink chamomile tea.	848	(86.5)	861	(87.9)	13	(1.3)	0.3443
It is when the doctor prescribes us remedies.	668	(68.2)	720	(73.5)	52	(5.3)	0.0026
It is when we take remedies without going to the doctor.*	479	(48.9)	566	(57.8)	87	(8.9)	< 0.001
I don’t know.	875	(89.3)	901	(91.9)	26	(2.7)	0.0303
**Schoolchildren chose the correct option and no other option(s)**	**454**	**(46.3)**	**532**	**(54.3)**	**78**	**(8.0)**	**< 0.001**
**Q8. What would you do to prevent antibiotic resistance? Mark with an X the correct answer**:							
Take the antibiotic that a relative advised me.	898	(91.6)	924	(94.3)	26	(2.7)	0.0140
Take the antibiotic I have at home.	919	(93.8)	941	(96.0)	22	(2.2)	0.0164
Consult a doctor and follow his instructions.*	719	(73.4)	751	(76.6)	32	(3.3)	0.0558
Take antibiotics every time I get sick.	836	(85.3)	908	(92.7)	72	(7.3)	< 0.001
I don’t know.	879	(89.7)	898	(91.6)	19	(1.9)	0.1045
**Schoolchildren chose the correct option and no other option(s)**	**619**	**(63.2)**	**731**	**(74.6)**	**112**	**(11.4)**	**< 0.001**

**Table legend**: This table shows the number and proportion of schoolchildren that answered correctly to the individual multiple-choice options of each question, that is, that correctly picked or did not pick a specific option at pretest and posttest, as well as the change from pre- to posttest. In the column Change (%), a positive value indicates an increase in correct answers in relation to the learning objectives of the Alforja, while a negative value indicates a decrease. A *p*-value below 0.05 was considered significant in this study. For each question, the row “Schoolchildren chose the correct option/s and no other option/s”, shows the number and proportion of schoolchildren that answered the overall questions correctly. Schoolchildren’s responses to the overall questions were considered correct if they had chosen the correct multiple-choice option(s) and no other options. While schoolchildren may have responded correctly to one or more multiple-choice option(s), they may have also picked other option(s) incorrectly, and hence the overall answer is not a sum of the individual multiple-choice options. The option “Other” in Q1, Q5, Q7, and Q8 was omitted from the table as almost no schoolchildren chose this option. Also, Q4 was omitted from analysis as it was not a knowledge-based question

*Correct multiple-choice option(s). Q1, Q2, Q5, Q7 and Q8 had only one correct option, while Q3 and Q6 had multiple correct options

There was no significant association between the change in total score (categorized as decrease, no difference, increase) from pretest to posttest and gender (girls *vs.* boys, *p* = 0.093), nor school type (private *vs.* public schools, *p* = 0.15). However, there was a significant correlation with geographical area (urban *vs.* rural, *p* = 0.006), with a greater, yet modest, proportion of schoolchildren having an increased score in the urban area (Table 3).

**Table 3:** Total score change, pretest to posttest, in relation to gender, school type and geography (*N* = 980)

**Characteristic**	Decreased score		No difference		Increased score		*p*-value
	*n*	(%)	*n*	(%)	*n*	(%)	
Gender							
Male	64	(14.8)	88	(20.3)	281	(64.9)	0.093
Female	76	(13.9)	84	(15.4)	387	(70.7)	
School Type							
Public	118	(15.4)	130	(16.9)	520	(67.7)	0.150
Private	22	(10.4)	42	(19.8)	148	(69.8)	
Geography							
Urban	54	(10.8)	89	(17.8)	357	(71.4)	0.006
Rural	86	(17.9)	83	(17.3)	311	(64.8)	

## Discussion

The results from the questionnaire suggest that the Alforja Educativa was effective in increasing Ecuadorian schoolchildren’s knowledge on bacteria, antibiotic use, and antibiotic resistance. After participation in the Alforja program, there was a significant improvement in the mean total score. In addition, there was a significant positive change in the proportion of correct responses to six of the seven of the knowledge-based questions, and 23 of the 32 individual multiple-choice options.

The greatest improvements were seen in schoolchildren’s perceptions of bacteria. Before participation in the Alforja program, almost all schoolchildren had heard about bacteria, and the majority had learned about them from school. However, schoolchildren mainly saw bacteria as bad and associated them with causing illness. Only 37% were aware that bacteria can be both good and bad and just 14% had known that bacteria are important to life. These findings are aligned with previous research showing that the essential role of bacteria is often underappreciated [14, 15]. For example, in a study from Italy, fourth to eighth grade students considered microbes to be evil organisms, threatening their health and wellbeing, and only 25% of students recognized that bacteria can play a positive role [16]. The Alforja Educativa aims to shift negative perceptions about bacteria and promote a better understanding of their important role in nature in a multitude of processes. It is a major challenge in teaching to provide students with a nuanced understanding of microbes [15]. After participation in the Alforja program, most schoolchildren (86%) were able to identify that bacteria can be both good and bad, and 70% understood bacteria are important to life.

Furthermore, schoolchildren’s knowledge also increased on the meaning of antibiotic resistance, the meaning of self-medication, and how to avoid antibiotic resistance. At baseline, less than half of the school children were able to identify the meaning of antibiotic resistance, whereas this increased to three-quarters by post-test. Knowledge about self-medication had a moderate increase, however, still only about half of the schoolchildren could identify the correct meaning of the word after participating in the Alforja program. At the same time, already before starting the Alforja program, most schoolchildren understood that to prevent antibiotic resistance they should not take antibiotics that a relative advised, one they have at home or take antibiotics every time they get sick. This suggests the terminology of “self-medication” might be advanced for schoolchildren, and future work should consider the use of age-appropriate terminology [17].

When asked about the correct use of antibiotics, the individual multiple-choice options had mixed results, leading to no change in the overarching question. While there was an increased understanding that antibiotics should not be used to treat a cold, there was a decreased understanding that antibiotics are used to treat illness caused by bacteria. At follow-up, almost all schoolchildren understood that antibiotic treatment should not be stopped as soon as the person feels better. Hence it seems there was confusion amongst the children in relation to some of these topics. It is possible that certain themes came across better in the program (such as not using antibiotics for colds), while other concepts need more explanation (such as when and for what diseases antibiotics should be used). It is also possible that the questions and answer options were posed in a way that was difficult to understand for the children (see limitation section).

In this study there were no significant differences in the scores of boys and girls or between public and private schools. However, schoolchildren in urban schools were more likely to score higher after the training than those in rural areas, the reason for this is unknown.

The findings of this study are aligned with previous research. A systematic review of the effectiveness of interventions to improve the public’s awareness of antimicrobial resistance identified six studies of educational interventions targeting schoolchildren. In all studies, a significant increase in knowledge occurred following the educational interventions [18]. One of the more well-studied interventions is e-Bug, a health education resource about antibiotic use and infection prevention that has been rolled out in a number of countries foremost in Europe. The implementation of e-Bug activities has shown to improve knowledge among participating children and their peers [19–22].

The Child-to-Child methodology of the Alforja Educativa gives schoolchildren an active role in their learning process, incentivizing them to learn through recreational, artistic and practical experiences [11, 23]. Not only does it create opportunities to improve knowledge among schoolchildren and their teachers but also for them to share with their families and communities [11, 23]. Milandri 2004, found that schoolchildren’s negative perceptions of bacteria and microbes stemmed from a general state of misunderstanding within the family and society and suggests that more precise information should be taught to schoolchildren, using them as link, to share information back to their families [16].

### Limitations

Several of the limitations of this study are related to the pretest-posttest tool to measure knowledge. With only seven questions the questionnaire may have been too short to provide a comprehensive understanding of the children’s knowledge on antibiotics and correct practices. In this study, the questionnaire was kept short considering the young age of the participants, but a longer questionnaire could be considered for future studies. In some cases, questions and answer options may have led the schoolchildren to pick the correct option without really understanding why. For example, in Q8 they may have picked “consult a doctor and follow his instructions” because doctors hold a position of authority and it is expected that we follow their advice. In choosing that option, the schoolchildren may not have also understood that the other answer options were incorrect and why they were not. Additionally, the combination of single and multiple answer questions, introduced challenges in data analysis and interpretation. The only question which did not have a significant change between pretest and posttest had eight multiple-choice options, three of which were correct responses. Likewise, the other question with two correct multiple-choice options had only a modest increase at follow-up. Also for the single answer questions, several children chose more than one option. This indicates that it may have been challenging for the schoolchildren to understand the questions with more than one correct multiple-choice option or that instructions to the schoolchildren were not clear enough. It is also possible that not all the themes came across clearly to the students during the Alforja program. Had a pilot been conducted before the study, some of these issues may have been discovered and possible to resolve. The short-comings of the pretest-posttest will be important to consider for revisions of the teaching materials and development of future questionnaires. Furthermore, in the present study, the time between pre- and posttest was about three months, and there was no longer-term follow-up on the schoolchildren’s knowledge. Finally, this study was limited to measuring knowledge, which is only one of several components influencing behavior, and it is well-known that knowledge alone is not enough to change beliefs, nor culture, attitudes or practices [24–26]. The Alforja Educativa program was developed with the aim to influence attitudes and behavior through the activities, co-creation and action agenda built into the program, and hence some of the knowledge questions asked about appropriate behaviors and practices when it comes to antibiotic use and resistance. However, further research on the impact of the Alforja Educativa on behaviors related to antibiotic use is warranted.

### Strengths

Despite these limitations, the study also has several strengths, including a large sample size with more than 1200 schoolchildren trained, and with close to 1000 schoolchildren that completed both pre- and posttest. The study was carried out in over 20 schools, both public and private, and in both urban and rural areas, thus covering a wide range of settings and schoolchildren. Furthermore, this intervention also included the training of more than 80 university students, exposing them to the same important information as the schoolchildren. The Alforja Educativa takes an innovative approach to addressing antibiotic resistance, placing it within a broader frame of health, well-being, and ecological awareness. An ecosystems approach to harmonious living is the basis for Sumak Kawsay, a concept deeply ingrained into the Andean culture and even part of the Ecuadorian constitution. During the pretest, over two-thirds of schoolchildren correctly identified the meaning of Sumak Kawsay, creating a natural foundation to explain the complex relationship between humans and bacteria, and the importance to use antibiotics with care to limit the development of antibiotic resistance. Context appropriate interventions are more likely to be effective in their implementation [4].

While the Alforja Educativa is intended for a Latin American audience, if tailored appropriately, it could also be suitable in other settings, especially those with indigenous principles valuing a balance in the environment or those who have a strong connection between the health of the planet, animals and people [27].

## Conclusions

Overall, implementation of the Alforja Educativa in schools in Ecuador improved schoolchildren’s knowledge on concepts related to bacteria, antibiotics and antibiotic resistance. There was a substantial positive shift in the schoolchildren’s perceptions of bacteria and understanding of their importance for life, health and well-being. The holistic perspective taken to explain the complex relationship between humans and bacteria, as well as the effect of antibiotics on the microbial world, may help provide a foundation for more sustainable antibiotic use.

### Electronic supplementary material

Below is the link to the electronic supplementary material.

Supplementary Material 1

## Data Availability

All data generated or analyzed during this study are available from the corresponding author on reasonable request.
