# Supplementary material for: Impact of the ´Alforja Educativa’ on Ecuadorian schoolchildren’s knowledge of bacteria, antibiotics, and antibiotic resistance, a pretest-posttest study
Source: BMC Public Health. 2024 Mar 18;24:844. doi: 10.1186/s12889-024-18340-0 (PMC10949744; doi:10.1186/s12889-024-18340-0)
Supplement: Supplementary file 1 — Supplementary Material 1 [file 12889_2024_18340_MOESM1_ESM.pdf]

Additional file 1. Alforja Educativa pretest-posttest questionnaire, English translation
For publication purposes only

CO-INVESTIGATOR DETAILS:<br>*Surname and Name: ___________________________University: ________________ Case No.___________*

ORGANIZATION, PARTICIPATION AND SOCIAL EMPOWERMENT PROGRAM

QUESTIONNAIRE

Name and Surname: ____

Age: ___________________________ Gender: Male ☐ Female ☐

School: ____

This questionnaire will not be graded, however, your answers are very important to help us improve. You need to pay attention and answer honestly. If you do not know the answer to a question, you can indicate "I don't know".

1. **What does "Sumak Kawsay" or "Good Living" mean? Mark with an X what you think is correct:**

- To have everything you need for life.
- To live in harmony between human beings and nature.
- To have a lot of money.
- Other _________________________________
- I don't know.

2. **Mark with an X the drawing that represents what bacteria are like:**

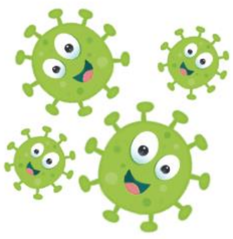

☐ **Good**

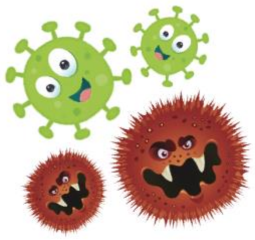

☐ **Good and Bad**

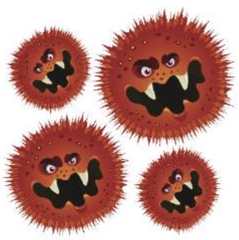

☐ **Bad**

3. **Mark with an X the correct answers:**

- Bacteria produce vitamins.
- Bacteria are important for life.
- Bacteria only serve to make us sick.
- I don't know.

4. **Where do you remember to have seen or heard something about bacteria? Mark the answer with an X. You can mark more than one option:**

- Radio
- Television
- School
- Supermarkets
- Home
- Nowhere
- Other _______________ (Write the place)

5. **What do you think antibiotic resistance means? Mark with an X the correct answer:**

- That bacteria are resistant to alcohol.
- That bacteria are resistant to soaps.
- That bacteria are resistant to antibiotics.
- Other ________________________________
- I don't know.

6. **Mark with an X what you consider correct about the use of antibiotics. It is possible to indicate more than one option:**

- Antibiotics are bought without a prescription.
- Antibiotics should always be prescribed by the doctor.
- Antibiotics are remedies that help you grow.
- Antibiotics are remedies to cure illnesses caused by bacteria.
- You can take antibiotics to treat a cold.
- Antibiotics should be taken as prescribed by the doctor.
- Antibiotics should be stopped as soon as the person feels better.
- I don't know.

7. **What does self-medication mean to you? Mark with an X the correct answer:**

- It is when we drink chamomile tea.
- It is when the doctor prescribes us remedies.
- It is when we take remedies without going to the doctor.
- Other ________________________________
- I don't know.

8. **What would you do to prevent antibiotic resistance? Mark with an X the correct answer:**

- Take the antibiotic that a relative advised me.
- Take the antibiotic I have at home.
- Consult a doctor and follow his instructions.
- Take antibiotics every time I get sick.
- Other ________________________________
- I don't know.

THANK YOU FOR YOUR COLLABORATION!
